# The ENIGMA Consortium: large-scale collaborative analyses of neuroimaging and genetic data

**DOI:** 10.1007/s11682-013-9269-5

**Published:** 2014-01-08

**Authors:** Paul M. Thompson, Jason L. Stein, Sarah E. Medland, Derrek P. Hibar, Alejandro Arias Vasquez, Miguel E. Renteria, Roberto Toro, Neda Jahanshad, Gunter Schumann, Barbara Franke, Margaret J. Wright, Nicholas G. Martin, Ingrid Agartz, Martin Alda, Saud Alhusaini, Laura Almasy, Jorge Almeida, Kathryn Alpert, Nancy C. Andreasen, Ole A. Andreassen, Liana G. Apostolova, Katja Appel, Nicola J. Armstrong, Benjamin Aribisala, Mark E. Bastin, Michael Bauer, Carrie E. Bearden, Ørjan Bergmann, Elisabeth B. Binder, John Blangero, Henry J. Bockholt, Erlend Bøen, Catherine Bois, Dorret I. Boomsma, Tom Booth, Ian J. Bowman, Janita Bralten, Rachel M. Brouwer, Han G. Brunner, David G. Brohawn, Randy L. Buckner, Jan Buitelaar, Kazima Bulayeva, Juan R. Bustillo, Vince D. Calhoun, Dara M. Cannon, Rita M. Cantor, Melanie A. Carless, Xavier Caseras, Gianpiero L. Cavalleri, M. Mallar Chakravarty, Kiki D. Chang, Christopher R. K. Ching, Andrea Christoforou, Sven Cichon, Vincent P. Clark, Patricia Conrod, Giovanni Coppola, Benedicto Crespo-Facorro, Joanne E. Curran, Michael Czisch, Ian J. Deary, Eco J. C. de Geus, Anouk den Braber, Giuseppe Delvecchio, Chantal Depondt, Lieuwe de Haan, Greig I. de Zubicaray, Danai Dima, Rali Dimitrova, Srdjan Djurovic, Hongwei Dong, Gary Donohoe, Ravindranath Duggirala, Thomas D. Dyer, Stefan Ehrlich, Carl Johan Ekman, Torbjørn Elvsåshagen, Louise Emsell, Susanne Erk, Thomas Espeseth, Jesen Fagerness, Scott Fears, Iryna Fedko, Guillén Fernández, Simon E. Fisher, Tatiana Foroud, Peter T. Fox, Clyde Francks, Sophia Frangou, Eva Maria Frey, Thomas Frodl, Vincent Frouin, Hugh Garavan, Sudheer Giddaluru, David C. Glahn, Beata Godlewska, Rita Z. Goldstein, Randy L. Gollub, Hans J. Grabe, Oliver Grimm, Oliver Gruber, Tulio Guadalupe, Raquel E. Gur, Ruben C. Gur, Harald H. H. Göring, Saskia Hagenaars, Tomas Hajek, Geoffrey B. Hall, Jeremy Hall, John Hardy, Catharina A. Hartman, Johanna Hass, Sean N. Hatton, Unn K. Haukvik, Katrin Hegenscheid, Andreas Heinz, Ian B. Hickie, Beng-Choon Ho, David Hoehn, Pieter J. Hoekstra, Marisa Hollinshead, Avram J. Holmes, Georg Homuth, Martine Hoogman, L. Elliot Hong, Norbert Hosten, Jouke-Jan Hottenga, Hilleke E. Hulshoff Pol, Kristy S. Hwang, Clifford R. Jack, Mark Jenkinson, Caroline Johnston, Erik G. Jönsson, René S. Kahn, Dalia Kasperaviciute, Sinead Kelly, Sungeun Kim, Peter Kochunov, Laura Koenders, Bernd Krämer, John B. J. Kwok, Jim Lagopoulos, Gonzalo Laje, Mikael Landen, Bennett A. Landman, John Lauriello, Stephen M. Lawrie, Phil H. Lee, Stephanie Le Hellard, Herve Lemaître, Cassandra D. Leonardo, Chiang-shan Li, Benny Liberg, David C. Liewald, Xinmin Liu, Lorna M. Lopez, Eva Loth, Anbarasu Lourdusamy, Michelle Luciano, Fabio Macciardi, Marise W. J. Machielsen, Glenda M. MacQueen, Ulrik F. Malt, René Mandl, Dara S. Manoach, Jean-Luc Martinot, Mar Matarin, Karen A. Mather, Manuel Mattheisen, Morten Mattingsdal, Andreas Meyer-Lindenberg, Colm McDonald, Andrew M. McIntosh, Francis J. McMahon, Katie L. McMahon, Eva Meisenzahl, Ingrid Melle, Yuri Milaneschi, Sebastian Mohnke, Grant W. Montgomery, Derek W. Morris, Eric K. Moses, Bryon A. Mueller, Susana Muñoz Maniega, Thomas W. Mühleisen, Bertram Müller-Myhsok, Benson Mwangi, Matthias Nauck, Kwangsik Nho, Thomas E. Nichols, Lars-Göran Nilsson, Allison C. Nugent, Lars Nyberg, Rene L. Olvera, Jaap Oosterlaan, Roel A. Ophoff, Massimo Pandolfo, Melina Papalampropoulou-Tsiridou, Martina Papmeyer, Tomas Paus, Zdenka Pausova, Godfrey D. Pearlson, Brenda W. Penninx, Charles P. Peterson, Andrea Pfennig, Mary Phillips, G. Bruce Pike, Jean-Baptiste Poline, Steven G. Potkin, Benno Pütz, Adaikalavan Ramasamy, Jerod Rasmussen, Marcella Rietschel, Mark Rijpkema, Shannon L. Risacher, Joshua L. Roffman, Roberto Roiz-Santiañez, Nina Romanczuk-Seiferth, Emma J. Rose, Natalie A. Royle, Dan Rujescu, Mina Ryten, Perminder S. Sachdev, Alireza Salami, Theodore D. Satterthwaite, Jonathan Savitz, Andrew J. Saykin, Cathy Scanlon, Lianne Schmaal, Hugo G. Schnack, Andrew J. Schork, S. Charles Schulz, Remmelt Schür, Larry Seidman, Li Shen, Jody M. Shoemaker, Andrew Simmons, Sanjay M. Sisodiya, Colin Smith, Jordan W. Smoller, Jair C. Soares, Scott R. Sponheim, Emma Sprooten, John M. Starr, Vidar M. Steen, Stephen Strakowski, Lachlan Strike, Jessika Sussmann, Philipp G. Sämann, Alexander Teumer, Arthur W. Toga, Diana Tordesillas-Gutierrez, Daniah Trabzuni, Sarah Trost, Jessica Turner, Martijn Van den Heuvel, Nic J. van der Wee, Kristel van Eijk, Theo G. M. van Erp, Neeltje E. M. van Haren, Dennis van ‘t Ent, Marie-Jose van Tol, Maria C. Valdés Hernández, Dick J. Veltman, Amelia Versace, Henry Völzke, Robert Walker, Henrik Walter, Lei Wang, Joanna M. Wardlaw, Michael E. Weale, Michael W. Weiner, Wei Wen, Lars T. Westlye, Heather C. Whalley, Christopher D. Whelan, Tonya White, Anderson M. Winkler, Katharina Wittfeld, Girma Woldehawariat, Christiane Wolf, David Zilles, Marcel P. Zwiers, Anbupalam Thalamuthu, Peter R. Schofield, Nelson B. Freimer, Natalia S. Lawrence, Wayne Drevets, the Alzheimer’s Disease Neuroimaging Initiative, EPIGEN Consortium, IMAGEN Consortium, Saguenay Youth Study (SYS) Group

**Affiliations:** 1 Imaging Genetics Center, Institute for Neuroimaging and Informatics, Keck School of Medicine, University of Southern California, 2001 N. Soto Street, Los Angeles, CA 90033 USA; 2 Genetic Epidemiology Laboratory, Queensland Institute of Medical Research, Brisbane, Australia; 3 Broad Institute of Harvard and MIT, Boston, MA USA; 4 Department of Human Genetics, Radboud University Medical Centre, Nijmegen, The Netherlands; 5 Donders Institute for Brain, Cognition and Behaviour, Department of Cognitive Neuroscience, Radboud University Medical Centre, Nijmegen, The Netherlands; 6 Human Genetics and Cognitive Functions, Institut Pasteur, Paris, France; 7 CNRS URA 2182 ‘Genes, synapses and cognition’, Institut Pasteur, Paris, France; 8 Sorbonne Paris Cité, Human Genetics and Cognitive Functions, Université Paris Diderot, Paris, France; 9 Olin Neuropsychiatry Research Center, Institute of Living, Hartford Hospital, Hartford, CT USA; 10 Department of Psychiatry, Yale University School of Medicine, New Haven, CT USA; 11 Department of Psychiatry and Psychotherapy, University of Greifswald, Greifswald, Germany; 12 NORMENT, KG Jebsen Centre for Psychosis Research, Oslo University Hospital and Institute of Clinical Medicine, University of Oslo, Oslo, Norway; 13 Department of Molecular and Cellular Therapeutics, Royal College of Surgeons in Ireland, Dublin 2, Ireland; 14 Department of Genetics, Texas Biomedical Research Institute, San Antonio, TX USA; 15 Department of Psychiatry, University of Iowa, Iowa City, IA USA; 16 Department of Neurology, David Geffen School of Medicine, UCLA, Los Angeles, CA USA; 17 Centre for Cognitive Ageing and Cognitive Epidemiology, The University of Edinburgh, 7 George Square, Edinburgh, UK; 18 Scottish Imaging Network, A Platform for Scientific Excellence (SINAPSE) Collaboration, Scotland, UK; 19 Brain Research Imaging Centre, The University of Edinburgh, Edinburgh, UK; 20 Max Planck Institute of Psychiatry, Munich, Germany; 21 The Mind Research Network, Albuquerque, NM USA; 22 Department of Biological Psychology, VU University, Neuroscience Campus, Amsterdam, The Netherlands; 23 Department of Cognitive Neuroscience, Radboud University Medical Centre, Donders Institute for Brain, Cognition and Behavior, Nijmegen, The Netherlands; 24 Brain Center Rudolf Magnus, University Medical Center Utrecht, Utrecht, The Netherlands; 25 Massachusetts General Hospital, Boston, MA USA; 26 Karakter Child and Adolescent Psychiatry University Center, Nijmegen, The Netherlands; 27 N. I. Vavilov Institute of General Genetics, Russian Academy of Sciences, Gubkin str. 3, Moscow, 119991 Russia; 28 Department of Psychiatry, University of New Mexico, Albuquerque, NM USA; 29 Department of Electrical and Computer Engineering, University of New Mexico, Albuquerque, NM USA; 30 Clinical Neuroimaging Laboratory, National University of Ireland Galway, University Road, Galway, Ireland; 31 Center for Neurobehavioral Genetics, University of California, Los Angeles, CA USA; 32 The Kimel Family Translational Imaging Genetics Laboratory, The Centre for Addiction and Mental Health, Toronto, ON Canada; 33 Dr Einar Martens Research Group for Biological Psychiatry, Department of Clinical Medicine, University of Bergen, Bergen, Norway; 34 Center for Medical Genetics and Molecular Medicine, Haukeland University Hospital, Bergen, Norway; 35 Department of Genomics, Life and Brain Center, University of Bonn, Bonn, Germany; 36 Institute of Human Genetics, University of Bonn, Bonn, Germany; 37 Institute for Neuroscience and Medicine (INM-1), Centre Jülich, Jülich, Germany; 38 Division of Medical Genetics, Department of Biomedicine, University of Basel, Basel, Switzerland; 39 Department of Psychiatry, Marqués de Valdecilla University Hospital, IFIMAV, School of Medicine, University of Cantabria, Santander, Spain; 40 Centro Investigación Biomédica en Red Salud Mental (CIBERSAM), Madrid, Spain; 41 Department of Psychology, The University of Edinburgh, Edinburgh, UK; 42 Department of Neurology, Hopital Erasme, Universite Libre de Bruxelles, 1070 Brussels, Belgium; 43 School of Psychology, University of Queensland, Brisbane, QLD 4072 Australia; 44 Department of Medical Genetics, Oslo University Hospital, Oslo, Norway; 45 Neuropsychiatric Genetics Research Group, Department of Psychiatry, Institute for Molecular Medicine and Trinity College Institute for Neuroscience, Trinity College, Dublin, Ireland; 46 MGH/HMS Martinos Center for Biomedical Imaging, Massachusetts General Hospital, Charlestown, MA USA; 47 University Hospital C.G. Carus, Department of Child and Adolescent Psychiatry, Dresden University of Technology, Dresden, Germany; 48 Department of Psychiatry and Psychotherapy, Charité, Universitaetsmedizin Berlin, Charitè Campus Mitte, Berlin, Germany; 49 Department of Psychology, University of Oslo, Oslo, Norway; 50 Max Planck Institute for Psycholinguistics, 6500 AH Nijmegen, The Netherlands; 51 Research Imaging Institute, UT Health Science Center at San Antonio, San Antonio, TX USA; 52 South Texas Veterans Health Care Center, San Antonio, TX USA; 53 Neurospin, Commissariat à l’Energie Atomique, Paris, France; 54 Department of Psychiatry, Massachusetts General Hospital, Boston, MA USA; 55 German Center for Neurodegenerative Diseases (DZNE), University of Greifswald, Greifswald, Germany; 56 Central Institute of Mental Health, Medical Faculty Mannheim, University of Heidelberg, Mannheim, Germany; 57 Center for Translational Research in Systems Neuroscience and Psychiatry, Department of Psychiatry, Georg August University, Goettingen, Germany; 58 Division of Psychiatry, Royal Edinburgh Hospital, University of Edinburgh, Edinburgh, UK; 59 Department of Molecular Neuroscience, UCL Institute, London, UK; 60 Department of Diagnostic Radiology and Neuroradiology, University of Greifswald, Greifswald, Germany; 61 Department of Psychiatry, University Medical Center Groningen, University of Groningen, Groningen, The Netherlands; 62 Interfaculty Institute for Genetics and Functional Genomics, University of Greifswald, Greifswald, Germany; 63 National Institute of Health Research Biomedical Research Centre for Mental Health, South London and Maudsley National Health Service Foundation Trust, London, UK; 64 King’s College London, Institute of Psychiatry, London, UK; 65 Department of Clinical Neuroscience, Karolinska Institutet and Hospital, Stockholm, Sweden; 66 Department of Clinical and Experimental Epilepsy, UCL Institute of Neurology, London, UK; 67 Center for Computational Biology and Bioinformatics, Indiana University School of Medicine, Indianapolis, IN USA; 68 Department of Radiology and Imaging Sciences, Center for Neuroimaging, Indiana University School of Medicine, Indianapolis, IN USA; 69 Maryland Psychiatric Research Center, Department of Psychiatry, University of Maryland School of Medicine, Baltimore, MD USA; 70 Psychiatric and Neurodevelopmental Genetics Unit, Center for Human Genetic Research, Massachusetts General Hospital, Boston, MA USA; 71 Mood and Anxiety Disorders Section, Human Genetics Branch, Intramural Research Program, National Institute of Mental Health, National Institutes of Health, US Dept of Health and Human Services, Bethesda, MD USA; 72 Taub Institute for Research on Alzheimer Disease and the Aging Brain, Columbia University Medical Center, New York, NY USA; 73 MRC-SGDP Centre, Institute of Psychiatry, King’s College London, London, UK; 74 Department of Psychiatry and Human Behavior, University of California, Irvine, CA USA; 75 Centre for Healthy Brain Ageing, School of Psychiatry, University of New South Wales Medicine, Sydney, New South Wales Australia; 76 Department of Biomedicine, Aarhus University, Aarhus, Denmark; 77 Department of Genomic Mathematics, University of Bonn, Bonn, Germany; 78 Research Unit, Sorlandet Hospital HF, Kristiansand, Norway; 79 Centre for Advanced Imaging, University of Queensland, Brisbane, Australia; 80 Ludwig-Maximilians-University (LMU), Munich, Germany; 81 Department of Psychiatry and Neuroscience Campus Amsterdam, VU University Medical Center, Amsterdam, The Netherlands; 82 Centre for Genetic Origins of Health and Disease, The University of Western Australia, Perth, Australia; 83 Department of Psychiatry, University of Minnesota Medical Center, Minneapolis, MN USA; 84 Department of Psychology, Stockholm University, Stockholm, Sweden; 85 Umeå Center for Functional Brain Imaging (UFBI), Umeå University, Umeå, Sweden; 86 Department of Psychiatry, UT Health Science Center at San Antonio, San Antonio, TX USA; 87 Rotman Research Institute, University of Toronto, Toronto, ON Canada; 88 The Hospital for Sick Children, University of Toronto, Toronto, ON Canada; 89 Department of Psychiatry and Leiden Institute for Brain and Cognition, Leiden University Medical Center, Leiden, The Netherlands; 90 Department of Medical and Molecular Genetics, King’s College London, London, UK; 91 Neuropsychiatric Institute, Prince of Wales Hospital, Sydney, New South Wales Australia; 92 Laureate Institute for Brain Research, Tulsa, OK USA; 93 Department of Medical and Molecular Genetics, Indiana University School of Medicine, Indianapolis, IN USA; 94 Department of Psychiatry, Beth Israel Deaconess Medical Center, Boston, MA USA; 95 Department of Neuroimaging, Institute of Psychiatry, King’s College London, London, UK; 96 NIHR Biomedical Research Centre for Mental Health at South London and Maudsley NHS Trust and Institute of Psychiatry, King’s College London, London, UK; 97 Minneapolis VA Health Care System, Minneapolis, MN USA; 98 Behavioural and Cognitive Neuroscience Neuroimaging Center, University Medical Center Groningen, Groningen, The Netherlands; 99 Institute for Community Medicine, University of Greifswald, Greifswald, Germany; 100 Departments of Radiology, Medicine, Psychiatry, University of California, San Francisco, CA USA; 101 Department of Child and Adolescent Psychiatry, Erasmus University Medical Centre, Rotterdam, The Netherlands; 102 Radboud University NijmegenDonders Institute for Brain, Cognition and Behavior, Centre for Cognitive Neuroimaging, Nijmegen, The Netherlands; 103 Oxford Centre for Functional MRI of the Brain (FMRIB), University of Oxford, Oxford, UK; 104 Neuroscience Research Australia, Sydney, Australia; 105 School of Medical Sciences, University of New South Wales, Sydney, Australia; 106 Center for Brain Science, Harvard University, Cambridge, MA USA; 107 The Brain and Mind Research Institute, University of Sydney, Sydney, Australia; 108 Alzheimer Scotland Dementia Research Centre, University of Edinburgh, Edinburgh, UK; 109 Centre for Regenerative Medicine, University of Edinburgh, Edinburgh, UK; 110 Centre for Clinical Brain Sciences, The University of Edinburgh, Edinburgh, UK; 111 Department Early Psychosis, Academic Psychiatric Centre, AMC, UvA, Amsterdam, Netherlands; 112 EMGO + Institute, VU University Medical Center, Amsterdam, The Netherlands; 113 Cognitive Science Department, UC San Diego, La Jolla, CA USA; 114 Department of Psychiatric Research, Diakonhjemmet Hospital, Oslo, Norway; 115 Department of Clinical Neuropsychology, VU University, Amsterdam, The Netherlands; 116 Reta Lila Weston Institute and Department of Molecular Neuroscience, UCL Institute of Neurology, London, UK; 117 Department of Neurology and NeuroSurgery, McGill University, Montreal, Quebec Canada; 118 Department of Psychiatry, University of Pittsburgh, Pittsburgh, PA USA; 119 Department of Psychiatry, Dalhousie University, Halifax, Nova Scotia Canada; 120 Department of Psychiatry, University of Oxford, Oxford, UK; 121 Department of Psychiatry and Behavioral Sciences, University of Texas Medical School, Houston, TX USA; 122 University of Texas Center of Excellence on Mood Disorders, Department of Psychiatry, UT Medical School, Houston, TX USA; 123 Department of Psychiatry and Biobehavioral Sciences and the Center for Neurobehavioral Genetics, The Semel Institute for Neuroscience and Human Behavior, UCLA, Los Angeles, CA USA; 124 Longitudinal Studies Section, Translational Gerontology Branch, National Institute on Aging, Baltimore, MD USA; 125 Berlin School of Mind and Brain, Humboldt University Berlin, Berlin, Germany; 126 Department of Psychiatry and Psychotherapy, Carl Gustav Carus University Hospital, Dresden, Germany; 127 Department of Psychiatry and Psychotherapy, Helios Hospital Stralsund, Stralsund, Germany; 128 Department of Psychiatry, Harvard Medical School, Harvard University, Cambridge, MA USA; 129 Department of Psychiatry, Brown University, Providence, RI USA; 130 Psychosis Research Unit, Mount Sinai School of Medicine, New York, NY USA; 131 Department of Psychiatry and Behavioral Neuroscience, University of Cincinnati College of Medicine, Cincinnati, OH USA; 132 Department of Psychiatry, University of Pennsylvania, Philadelphia, PA USA; 133 Philadelphia Veterans Administration Medical Center, Philadelphia, PA USA; 134 Department of Psychiatry and Psychotherapy, University Regensburg, Regensburg, Germany; 135 Department of Psychiatry and Psychotherapy, Trinity College, University Dublin, Dublin, Germany; 136 Stockholm Brain Institute, Stockholm, Sweden; 137 Department of Psychology and Neuroscience Institute, Georgia State University, Atlanta, GA USA; 138 Department of Psychology, Neuroscience and Behaviour, McMaster University, Hamilton, ON Canada; 139 Department of Psychology, University of New Mexico, Albuquerque, NM USA; 140 Department of Psychosomatic Medicine, Oslo University Hospital, Oslo, Norway; 141 Institute of Clinical Medicine, University of Oslo, Oslo, Norway; 142 Indiana Alzheimer Disease Center, Indiana University School of Medicine, Indianapolis, IN USA; 143 Department of Radiology, University of Calgary, Calgary, Alberta Canada; 144 Department of Statistics & Warwick Manufacturing Group, The University of Warwick, Coventry, UK; 145 Departments of Psychiatry and Behavioral Sciences and Radiology, Northwestern University, Chicago, IL USA; 146 Electrical Engineering, Vanderbilt University, Nashville, TN USA; 147 Experimental Therapeutics and Pathophysiology Branch, National Institute of Mental Health, Bethesda, MD USA; 148 Institute of Clinical Chemistry and Laboratory Medicine, University of Greifswald, Greifswald, Germany; 149 Institute of Neuroscience and Physiology, University of Gothenburg, Gothenburg, Sweden; 150 Department of Medical Epidemiology and Biostatistics, Karolinska Institutet, Stockholm, Sweden; 151 Institute of Biomaterials and Biomedical Engineering, University of Toronto, Toronto, ON Canada; 152 Faculty of Community Medicine, University of Tulsa, Tulsa, OK USA; 153 Maryland Institute for Neuroscience and Development (MIND), Chevy Chase, MD USA; 154 Mathison Centre for Mental Health Research and Education, Hotchkiss Brain Institute, University of Calgary, Calgary, Alberta Canada; 155 Munich Cluster for Systems Neurology (SyNergy), Munich, Germany; 156 MRC Centre for Neuropsychiatric Genetics and Genomics, Institute of Psychological Medicine and Clinical Neurosciences, Cardiff University, Cardiff, UK; 157 Neuroscience and Mental Health Research Institute, Cardiff University, Cardiff, UK; 158 School of Medical Sciences, University of New South Wales, Kensington, NSW Australia; 159 Oakland University William Beaumont School of Medicine, Rochester Hills, MI USA; 160 CHU Sainte Justine University Hospital Research Center, Montreal, QC Canada; 161 Addictions Department, King’s Health Partners, King’s College London, London, UK; 162 South Texas Veterans Health Care System, San Antonio, TX USA; 163 Research Unit 1000, Neuroimaging and Psychiatry, INSERM-CEA-Faculté de Médecine Paris Sud University-Paris Descartes University, Maison de Solenn Paris, SHFJ Orsay, Paris, France; 164 Department of Genetics, King Faisal Specialist Hospital and Research Centre, Riyadh, Saudi Arabia; 165 Department of Psychiatry, Rudolf Magnus Institute, University Medical Center Utrecht, Utrecht, The Netherlands; 166 School of Mathematics and Statistics, University of Sydney, Sydney, Australia; 167 School of Medicine, University of Nottingham, Nottingham, UK; 168 Department of Psychiatry, Stanford University School of Medicine, Stanford, CA USA; 169 Aging Research Center, Karolinska Institutet and Stockholm University, Stockholm, Sweden; 170 Hellen Wills Neuroscience Institute, Brain Imaging Center, University of California at Berkeley, Berkeley, CA USA; 171 Department of Psychiatry, University of Missouri, Columbia, MO USA; 172 Departments of Psychiatry and Neurobiology, Yale University School of Medicine, New Haven, CT USA; 173 Mayo Clinic, Rochester, MN USA; 174 Transdisciplinary and Translational Prevention Program, RTI International, Baltimore, MD USA; 175 Department of Psychiatry and Neuroscience, Icahn School of Medicine at Mount Sinai, New York, NY USA; 176 Department of Psychiatry, University of Halle, Halle, Germany; 177 Advanced Biomedical Informatics Group, llc., Iowa City, IA USA; 178 Program in Neurogenetics, Department of Neurology, David Geffen School of Medicine, University of California Los Angeles, Los Angeles, CA 90095 Netherlands; 179 QIMR Berghofer Medical Research Institute, Quantitative Genetics, Brisbane, Australia; 180 QIMR Berghofer Medical Research Institute, Genetic Epidemiology, Brisbane, Australia; 181 QIMR Berghofer Medical Research Institute, Neuroimaging Genetics, Brisbane, Australia; 182 Department of Psychology, UCLA, Los Angeles, CA USA; 183 Department of Pharmacological and Biomolecular Sciences, University of Milan, Milan, Italy; 184 Dr. E. Martens Research Group for Biological Psychiatry, Center for Medical Genetics and Molecular Medicine, Haukeland University Hospital, Bergen, Norway; 185 Department of Psychiatry, UHC University of Vermont, Bergen, VT USA; 186 Centre for Healthy Brain Ageing, Psychiatry, University of New South Wales (UNSW), Sydney, Australia; 187 Department of Psychiatry and Biobehavioral Sciences, UCLA School of Medicine, Los Angeles, CA USA; 188 School of Psychology, University of Exeter, Exeter, UK; 189 Janssen Research & Development, of Johnson & Johnson, Inc., 1125 Trenton-Harbourton Road, Titusville, NJ 08560 USA

**Keywords:** Genetics, MRI, GWAS, Consortium, Meta-analysis, Multi-site

## Abstract

The Enhancing NeuroImaging Genetics through Meta-Analysis (ENIGMA) Consortium is a collaborative network of researchers working together on a range of large-scale studies that integrate data from 70 institutions worldwide. Organized into Working Groups that tackle questions in neuroscience, genetics, and medicine, ENIGMA studies have analyzed neuroimaging data from over 12,826 subjects. In addition, data from 12,171 individuals were provided by the CHARGE consortium for replication of findings, in a total of 24,997 subjects. By meta-analyzing results from many sites, ENIGMA has detected factors that affect the brain that no individual site could detect on its own, and that require larger numbers of subjects than any individual neuroimaging study has currently collected. ENIGMA’s first project was a genome-wide association study identifying common variants in the genome associated with hippocampal volume or intracranial volume. Continuing work is exploring genetic associations with subcortical volumes (ENIGMA2) and white matter microstructure (ENIGMA-DTI). Working groups also focus on understanding how schizophrenia, bipolar illness, major depression and attention deficit/hyperactivity disorder (ADHD) affect the brain. We review the current progress of the ENIGMA Consortium, along with challenges and unexpected discoveries made on the way.

## Introduction

### Origins of brain imaging in human populations

During the “Decade of the Brain” in the 1990s (Jones and Mendell 1999), a number of major neuroimaging centers began to scan hundreds of patients and healthy individuals using a variety of neuroimaging methods. The accelerating pace of data collection was driven mainly by the wide availability of MRI around the world. The structure and function of the living brain was beginning to be mapped in unprecedented detail in human populations.

In a typical neuroimaging study—both now and 20 years ago—between ten and a few hundred subjects might have been scanned, and statistical models would be fitted to identify factors that affect brain structure and function. Early studies—such as lesion studies—correlated radiological measures with clinical diagnosis and behavior, but the study of large populations represented a new movement in human brain mapping. Fundamental questions in neuroscience could now be examined—what are the effects of aging, degenerative disease and psychiatric illness on the living brain? How do brain measures relate to cognition and behavior? Do brain measures predict our risk for disease, or prognosis in those who are ill?

There was growing confidence that questions of broad societal and medical impact could be better understood if enough brain scans were collected—projects were initiated to examine effects on the brain of psychiatric medications, drugs and alcohol abuse, dietary factors, and many other factors including education, cardiovascular fitness, as well as pharmacologic and behavioral interventions.

At the same time, the broad availability of brain scans led to the development of widely adopted tools to analyze the resulting data. Software such as Statistical Parametric Mapping (SPM; Friston et al. 1995; Frackowiak 1997), FSL (Jenkinson et al. 2012), BRAINS (Pierson et al. 2011) and FreeSurfer (Fischl et al. 2004) among many other tools, were widely distributed over the internet. This made it feasible to analyze neuroimaging data and compute standardized measures from brain scans in a consistent and agreed way, albeit with methods that are continually refined.

### Early neuroimaging consortia

Early consortium efforts in neuroimaging included the International Consortium for Brain Mapping (ICBM; Mazziotta et al. 1995), which recognized the need to establish normative data on the brain from a wide range of human populations scanned in different parts of the world. The ICBM began with an effort to scan around 150 healthy subjects in Los Angeles, Montreal, and San Antonio, Texas, and grew to include sites in Europe and Asia that broadened the age range and ethnic groups assessed. Later, the ICBM also extended the depth of the neuroimaging measures to include functional MRI and even *post mortem* histology and cytoarchitecture (Amunts et al. 1999).

Given the wide variations in brain anatomy even among healthy subjects, consortia such as the ICBM developed a range of “average” anatomical templates based on MRI scans of hundreds of healthy subjects. Analysis software for brain images disseminated these average brain templates, and provided methods to relate new data to previously compiled atlases and data collections. This led to the notion of statistical representations of imaging signals in standardized coordinate spaces—or “statistical parametric maps”. The wide adoption of these standard spaces—such as the ICBM or MNI (Montreal Neurological Institute) spaces—was eased by the development of automated registration and alignment methods (Woods et al. 1993; Collins et al. 1994; Ashburner et al. 1999; Jenkinson et al. 2002) that allowed researchers to rapidly align their own data to the templates. This effort led to the rise of voxel-based morphometric approaches and statistical mapping approaches in general. These developments also allowed any group to compare and contrast their findings with ongoing findings from other groups around the world—a movement that was stimulated by the development of the Talairach and Tournoux brain atlases, which defined anatomical regions in stereotaxic space (Talairach et al. 1993). The Talairach atlas was among the first to compile a coordinate-based reference system, and it allowed researchers worldwide to relate their findings to existing data collections. In the mid-1990s, a group in San Antonio developed the “Talairach Daemon”, allowing electronic pooling of findings from brain mapping studies based on their coordinates in Talairach space. In addition to the use of standard anatomical templates for reporting results, this coordinate system opened the door for clinically-oriented consortia to scan and analyze large-scale patient populations in a consistent way. The rapid development of nonlinear registration methods also made it possible to improve the alignment of new datasets to digital anatomical templates, for coordinate based reporting of results.

The Alzheimer’s Disease Neuroimaging Initiative (ADNI), for example (Weiner et al. 2012), scanned around 800 people in its first phase, including healthy elderly people, individuals with mild cognitive impairment and patients with Alzheimer’s disease. ADNI began in 2005, after testing the feasibility and reproducibility of a range of scanning protocols. This led to standardized scanning methods implemented at 58 sites across North America (Leow et al. 2009; Jahanshad et al. 2010; Jack 2012; Zhan et al. 2012). Many other neuroimaging consortia have been established, including the functional Brain Imaging Research Network (FBIRN) (Potkin and Ford 2009) which has developed standardized methods for multi-center functional MRI studies (Glover et al. 2012) and the Mind Clinical Imaging Consortium (Gollub et al. 2013) focusing on schizophrenia, as well as research networks focusing on pediatric imaging (Evans 2006), autism (Ecker et al. 2013), HIV/AIDS (Cohen et al. 2010) and many others. In fact, the successes of these multi-site initiatives have led to large-scale neuroimaging efforts being initiated and funded in other countries (Carrillo et al. 2012; Alzheimer’s Association 2013; White et al. 2013).

### Genome-wide association studies (GWAS)

At the same time, a number of *genetic* studies using twin or family-based designs had shown that many brain-derived measures were significantly heritable (Thompson et al. 2001; Baaré et al. 2001; White et al. 2002; Wright et al. 2002; van Erp et al. 2004a, b; Hulshoff Pol et al. 2006; Winkler et al. 2010; Kochunov et al. 2010; Blokland et al. 2012; Koten et al. 2009). In other words, a substantial fraction of the variability in brain measures—especially structural but also some functional measures, and even brain metabolites (Batouli et al. 2012)—could be explained by genetic relationships among individuals. The total amount of gray and white matter in the brain, the overall volume of the brain—and even activation patterns on fMRI or connections tracked with diffusion MRI—were more similar among family members than unrelated individuals (Peper et al. 2007; Koten et al. 2009; Glahn et al. 2010; Brouwer et al. 2010; Fornito et al. 2011; Blokland et al. 2012; Jahanshad et al. 2013a; Thompson et al. 2013; Van den Heuvel et al. 2013).

Arguably, it is equally important to identify regions or measures with low heritability as well. The reliability of imaging measures varies considerably by region or network, and so does the ability to detect heritability, even if present. Such information is immensely useful in constraining the potential phenotypes worth pursuing and interpreting results; we consider this further below.

Despite the high heritability of many brain measures (h^2^ up to 0.89; Kremen et al. 2009; or even up to 0.96: van Soelen et al. 2012), the specific genetic variants that contribute to this variability remain largely unknown. A possible exception is the Alzheimer’s disease (AD) risk gene, *APOE*: carriers of one or more risk-conferring alleles (*APOE4*) demonstrate accelerated gray matter loss with age (Lu et al. 2011). They also have a roughly three-fold increased risk for late-onset AD, for each risk allele they carry (Corder et al. 1993). In a recent meta-analysis of 35 prospective cohort studies with an average follow-up of 2.9 years, the odds ratio for conversion from mild cognitive impairment to Alzheimer’s dementia in *APOE4* carriers was determined to be 2.29, relative to non-carriers (Elias-Sonnenschein et al. 2011). Other prior papers reported a higher odds ratio, around 4 for heterozygotes and >7 for homozygotes, with some differences depending on the ancestry of the cohort. According to another more recent review, one copy of ApoE4 increases risk by ~2.6–3.4, and homozygotes for ApoE4 have an odds ratio of 14.9 compared to the reference genotype of E3/3 (Liu et al. 2013).

**Fig. 1:**
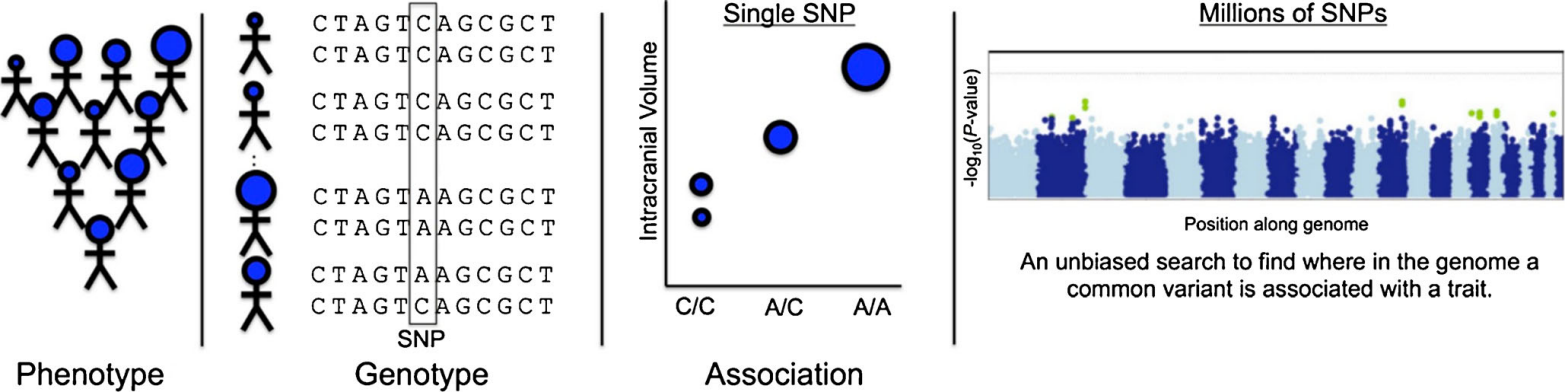
Steps involved in a genome-wide association study. A heritable brain measure (or “phenotype”) - which could be binary, such as a disease state, or continuous, such as the intracranial volume (ICV) - is extracted from brain imaging scans from a large group of people. To determine if there is any statistical association between this brain measure and the inter-subject variations at a single SNP, the genetic variations among individuals can be assessed at a single location along the genome, and correlated with differences in the trait of interest (here, ICV). Genome-wide association scans involve an unbiased search across the whole genome to discover novel genetic loci associated with the trait. Testing a million or more SNPs requires a strict multiple comparisons correction threshold, to avoid reporting spurious results; normally, credible findings have to achieve a significance value more extreme than *p* < 10^−8^. The so-called “Manhattan plot” on the right (by analogy with the Manhattan skyline in New York) displays the −log_10_ of the *p*-value for associations between the brain measure and genetic variation at each position along the genome; the higher the point on the plot, the more likely it is that an association exists. Of course, it is important not to see genome-wide significance as a “binary state”, whose conditions are either fulfilled or not—but rather a measure of the level of evidence for a genetic association. Findings in these plots must typically be replicated in several independent cohorts before they are considered credible or generalizable

A number of groups around the world began to perform GWAS on measures derived from brain images, with the goal of finding new genetic variants that might account for more of the variation in brain structure and function, and also for disease risk. The genetic variants of interest in a GWAS are single nucleotide polymorphisms, or SNPs, commonly carried variants in the genetic code. SNPs are DNA sequence variations that occur when a single nucleotide (A, G, C, or T) is altered; SNPs are thought to be point mutations that were not so damaging that evolution allowed them to be retained in a significant proportion of the population of a species. Within a population, SNPs can be assigned a minor allele frequency—the proportion of chromosomes in the population carrying the less common variant. Figure 1 illustrates the ideas behind this approach. Before we discuss GWAS, it is worth noting a distinction between narrow and broad-sense heritability: broad-sense heritability is the proportion of variation in a phenotype (here, individual variations in brain measures) that can be explained by genetic effects. These effects may include dominance and epistasis—interactions between SNPs or genes in different parts of the genome. The narrow-sense heritability is the proportion of variance in a brain measure that is accounted for by additive genetic factors (and this is typically a smaller proportion of the trait variance). These additive genetic effects are the types of statistical effects that GWAS aims to detect.

In a typical GWAS analysis, one might test ~2.5 million common SNPs in the genome, to see if any of these genetic variants are associated with a trait, such as a brain-derived measure, or a specific disease such as AD. Although not the only important type of genetic variation, SNPs can be measured using readily available genotyping arrays, and individually provide adequate statistical power as the variants are common enough to test their effects statistically. Because only selected SNPs capturing the variability of the genome are genotyped, many authors have argued that this genotyping technology is much less expensive than whole-genome sequencing. However, new technologies using low coverage sequencing with imputation may in some cases yield several times the effective sample size of GWAS based on SNP array data, and a commensurate increase in statistical power as described in Pasaniuc et al. (2012).

GWAS has had many successes. Many common polymorphisms have now been found that increase genetic risk for AD (Harold et al. 2009; Lambert et al. 2009; Naj et al. 2011), age-related cognitive decline (Davies et al. 2012), schizophrenia (Almasy et al. 2008; Stefansson et al. 2009; Ripke et al. 2011; Rietschel et al. 2012), bipolar disorder (Sklar et al. 2011; Cichon et al. 2011) as well as obesity (Yang et al. 2012), alcohol drinking (Schumann et al. 2011), tobacco smoking (Thorgeirsson et al. 2008), cardiovascular disease (CARDIoGRAMplusC4D Consortium et al. 2013), osteoporosis (Estrada et al. 2012), prevalent psychiatric disorders (Cross-Disorder Group of the Psychiatric Genomics Consortium et al. 2013) and for many other traits and diseases.

Imaging may play a role in finding out how these genes create risk for illness through their impact on the brain, by comparing brain scans of carriers versus non-carriers. One such example is the *ZNF804A* story. A variant within *ZNF804A* was the first genome-wide significant SNP associated with risk for schizophrenia (O’Donovan et al. 2008). The function of this variant was initially not clear. Prominent papers later appeared (e.g., Esslinger et al. 2009) using imaging to establish disturbed connectivity as a neurogenetic risk mechanism for psychosis. They showed that some variant in *ZNF804A* (or some variations in linkage disequilibrium with them) must be functional in the human brain. This was one of many early studies to validate the intermediate phenotype strategy in psychiatry.

Ongoing work comparing genome-wide data from patients with AD and healthy elderly people had begun to unearth a growing set of new AD risk genes (Bertram 2009). By 2009, meta-analyses of GWAS from multiple elderly cohorts had implicated a trio of new AD risk genes—*CLU*, *CR1* and *PICALM* (Harold et al. 2009). Each of these appeared to affect AD risk by around 10–20 %, consistently, in cohorts around the world (Logue et al. 2011). Additional AD risk variants were rapidly discovered as GWAS expanded to more populations with dementia and healthy controls (Hollingworth et al. 2011).

A flurry of such studies occurred—some showed brain differences in Alzheimer’s disease risk gene carriers a full 50 years before AD typically strikes (Braskie et al. 2011; Bralten et al. 2011). Others showed a pattern of brain changes in unaffected carriers that resembled the “footprint” of Alzheimer’s disease in the living brain (Biffi et al. 2010; Erk et al. 2011, Rajagopalan et al. 2013). These findings will require follow-up but illustrate the potential of using neuroimaging measures to explore the effects of genetic variation.

But a much more adventurous goal provided the driving force behind the new and emerging fields of *imaging genomics*. This goal was to use neuroimaging data directly, to screen the genome for common variants that might affect the brain. In other words, rather than using the images in secondary studies of what disease risk genes do, images could be screened to discover important genetic associations. (Instead of imaging genetics, the somewhat interchangeable term “*imaging genomics*” is also used; genomics tends to refer to any method that directly assesses variation in the genome, as opposed to studies that may assess a single locus only, or simpler family studies that may not even analyze DNA). The growing computational power to screen very large neuroimaging datasets—for the purpose of extracting meaningful features from them—made this an interesting and achievable objective. Advocates of “imaging genetics”—the genetic analysis of brain images (Glahn et al. 2007; Turner et al. 2006)—suggested that it might even be more efficient to screen traits derived from brain images to provide endophenotypes for brain disorders.

The main motivation to screen brain images was to find some heritable measure of disease burden that might be closer to the underlying genetic effect than clinical diagnosis based on cognitive and clinical tests. The *endophenotype* hypothesis, long advocated by psychiatric geneticists such as Irving Gottesman (Gottesman and Gould 2003; Blangero 2004; Goldman 2012; White and Gottesman 2012; Kendler and Neale 2010) suggested that one might fruitfully apply genetic screening to any reliable and heritable biomarkers of a disease—measures from the blood or cerebrospinal fluid (CSF), or even from brain scans, which by now had become quite plentiful. The original definition of “endophenotype” for an illness or disorder (see Gottesman and Gould 2003) suggested that an endophenotype should (i) be associated with the illness/disorder of interest, (ii) be heritable, (iii) be state-independent, i.e., seen in people even when they do not show symptoms of the illness/disorder, (iv) co-segregate with illness/disorder within families, and (v) be observed in relatives of affected family members at a higher rate than in the general population.

The search for endophenotypes of disease, for genetic analysis, is related to the goal of finding biomarkers for AD or any psychiatric illness, although the quest for biomarkers pre-dated efforts to find endophenotypes of disease. In addition, biomarkers may not be stable, as they may change during the disease course. The term “biomarkers” has been used with many different meanings, but in general biomarkers are measures of disease burden that can be objectively quantified, ideally allowing more objective or earlier diagnosis, and making it easier to test the effects of treatment or prevention.

Advocates of using imaging for genetic analysis pointed to several advantages that imaging provides now, as well as several potential advantages that it could provide in the foreseeable future. First, neuroimaging can yield reproducible measures of brain structure and (perhaps to a lesser extent) brain function. Structural measures of the brain, from MRI, tend to have relatively high reproducibility across measurement methods, and are generally consistent with expert tracings of the same structures (see Supplement of Stein et al. 2012; many studies have investigated the reliability of measures from brain MRI, e.g., Pengas et al. 2009). In a recent GWAS analysis, Holmes et al. (2012) showed high reliability for automated brain volumes of hippocampus (*r* = .98), amygdala (*r* = .91), and intracranial volume (*r* = .99) for a cohort of data collected across investigators and matched scanners. Nugent et al. (2012) also studied the inter-scanner reliability of the FIRST software for segmentation (Patenaude et al. 2011), and found it to be high. However, it is overly optimistic to always expect high reproducibility from automated segmentations of brain MRI, and the reproducibility is region specific. For example, both FSL and FreeSurfer tend to do less well in segmenting small structures relative to larger structures. There are also differences in accuracy and reliability among different methods for automated segmentation of the brain (Shokouhi et al. 2011). Cortical thickness and other local gray matter density measures can show reduced reliability owing to sensitivity to image contrast variability, which becomes particularly challenging in multicenter studies (Schnack et al. 2010). The volumes of some structures, such as the caudate, may even show systematic biases in certain populations because of tissue class ambiguity that arises as a consequence of white matter degradation near gray matter structures. Some cortical regions are also difficult to delineate accurately due to the large intersubject variability. As noted below, one goal of ENIGMA has been to screen brain measures for reproducibility, heritability, and association with disease, to see which ones are likely to be promising for genetic analysis (we return to this topic below; see also Table 1).

**Table 1:** Selection of brain measures for genetic analysis. In ENIGMA, the brain measures chosen for analysis had to be feasible to measure consistently and efficiently at a large number of sites, according to agreed protocols (available at enigma.ini.usc.edu). As power is limited in GWAS, various tactics may be useful in the future to boost the power to find genetic associations. Some of these are categorized here

Power enhancement approach	Principle	Pros and cons
1. Enhance the dataset	Increase the sample size (some GWAS studies now assess 100,000+ subjects (Lango Allen et al. 2010; Speliotes et al. 2010))	Identifies variants with smaller effect sizes, but is more costly
	Increase genomic coverage/sequencing	Picks up rarer variants, but requires even more subjects for power of low frequency variants. Also is more costly than genotyping common SNPs through genotyping arrays, but the cost is rapidly decreasing
	Increase the range of phenotypes studied	May be able to find a high effect size phenotype, but also need to correct for the number of measures assessed, which may be large (e.g., in “voxel-based” GWAS; Stein et al. 2010; Ge et al. 2012); if too many are assessed, power is low
2. Data reduction	Focus on candidate SNPs/genes, candidate pathways, candidate phenotypes	Avoids heavy statistical correction, but may miss unexpected variants or phenotypes
2.1. Based on classical genetics principles	Heritability screening—remove or de-emphasise measures with low heritability	This may empower genomic screens of complex phenotypes (e.g., genome-wide connectome-wide screens; Jahanshad et al. 2013a), see ENIGMA-DTI (Jahanshad et al., Jahanshad et al. 2013b).
	Genetic Clustering—find parts of an image or 3D cortical surface with common underlying genetic determination	GWAS on the resulting “genetic clusters” appears to have higher power than standard voxel-based approaches (Chiang et al. 2011, 2012; Chen et al. 2011; 2012)
2.2. Based on relevance to disease	Endophenotype ranking value (ERV; Glahn et al. 2012), aims to rank biomarkers in terms of their promise as endophenotypes for any heritable illness.	Balances the strength of the genetic signal for the endophenotype and the strength of its relation to the disorder of interest
2.3. based on using multivariate statistics	Use multiple predictors in the genome or image or both (reviewed in Hibar et al. 2011a; Thompson et al. 2013; Meda et al. 2012); Sparse regression (Vounou et al. 2010, 2012; Ge et al. 2012; Silver et al. 2012), compressive sensing, parallel ICA, machine learning methods	Can search both the image and the genome simultaneously Difficult to apply to distributed remote datasets for meta-analysis and may be difficult to interpret the biological relevance of the signal
3. Multimodality approaches Exploit joint information in several imaging modalities or other biomarkers at the same time	Multimodal data fusion using ICA (Calhoun et al. 2009) Seemingly Unrelated Regression to pool information across simultaneous models (SUR; Jahanshad et al. 2013c)	More work required to analyse mutliple data modalities at once (e.g., anatomical MRI and DTI)

Second, measures of brain volume, integrity, receptor distribution, or chemical composition, might be more directly related to the function of genes—both genes whose function is unknown, and known candidate genes—such as growth factors, transcription factors, guidance molecules, or neurotransmitters and their transporters. Many of these had already been implicated in the risk for psychiatric illness, and imaging offered the opportunity to study differences in brain connectivity or function, in carriers of genetic variants associated with disease risk. In the future, advanced MRI methods such as [^1^H]MR spectroscopy could be used for population genetic studies of the brain’s chemical composition, or PET studies to measure receptor distribution, but work soon began in earnest on the genetic analysis of more common and widely available types of brain scans—MRI and DTI (Diffusion Tensor Imaging).

At the same time, some seasoned geneticists were skeptical about imaging genetics. Concerns were raised about the costs of image acquisition, relative to a standard psychiatric diagnostic test. For a study to be feasible, the cost of data collection must be borne in mind, regardless of which method is ultimately more efficient in discovering genes and mechanisms contributing to brain disease.

By 2009, a number of GWAS studies had been performed on neuroimaging data. Among the first studies to report a positive finding—a so-called “genome-wide hit”—was the report by (Potkin et al. 2009a) that identified a key genetic variant in *TOMM40*, in linkage disequilibrium with *APOE*, the known risk gene for AD. Using hippocampal atrophy as a quantitative phenotype in a genome-wide scan, they assessed 381 participants in the ADNI (Alzheimer’s Disease Neuroimaging Initiative) study, to identify SNPs for which there was an interaction between the genotype and diagnosis on the quantitative trait. Variants in *TOMM40* appeared to affect hippocampal volume differently in AD patients versus controls. Working with genetic-founder populations, some GWAS-based studies revealed genome-wide hits in relatively small samples; in the Saguenay Youth Study, genetic variations in the *KCTD8* region were associated with brain size in a community-based sample of adolescent girls (rs716890, *P* = 5.40 × 10^−9^); (Paus et al. 2012). Furthermore, genotype in the top hit (rs716890) interacted with prenatal exposure to maternal cigarette smoking vis-à-vis cortical area and cortical folding; in exposed girls only, this genotype explained ~21 % of variance in the cortical area (Paus et al. 2012).

The ADNI work analyzed a publicly available dataset (adni.loni.usc.edu) for which MRI scans and GWAS data could be freely downloaded from approximately 800 people. The ease of access of the ADNI data, and the principle of broad dissemination of the genomic data with fairly few restrictions led to over 100 genetic studies of the ADNI data, many of them using GWAS designs (Saykin et al. 2010).

The ADNI dataset stimulated work in imaging genetics, as evidenced by the large number of published papers using the data (all ADNI genetics studies from the period 2009–2012,are reviewed by Shen et al., 2013; *this volume*). Even so, data from other elderly cohorts is needed to determine how well ADNI’s genetic findings generalize to other non-selected cohorts. ADNI deliberately selected participants who are predominantly Caucasian, and relatively free from drug or alcohol abuse and vascular disease. Genetic associations detected in ADNI may be stronger or weaker, or even not supported at all, in cohorts with different ethnic composition, or with other co-morbid health conditions. Because of this, it is important to recognize that some genetic associations may depend on the cohort studied, diagnostic or demographic criteria, and we should not expect all true genetic associations to be detectable in all cohorts.

### Sample sizes and power for GWAS

As with other phenotypes, the first GWAS findings for imaging phenotypes were difficult to replicate, probably due to the limited availability of replication samples. Prior work had cautioned that, using the most standard kind of univariate analysis of SNP effects, very large sample sizes would be required—far larger than a typical neuroimaging study—to discover influential genetic variants, unless their effect sizes on the brain phenotypes being analyzed were unusually large (Potkin et al. 2009b). The power of a GWAS study depends on the number of null genetic variants assessed, the expected effect size of the genetic variant (typically less than 1 % of the trait variance), and the population frequency of the variant (Potkin et al. 2009b; Flint et al. 2010; Paus et al. 2012). The power obviously also depends on the genetic architecture of the trait—key factors are the large number of effectively independent LD regions in the genome (regions of linkage disequilibrium, with correlated SNPs) and the number of detectable causal loci per phenotype (which is typically small). The first series of studies identifying genetic markers for MRI phenotypes that replicated across independent samples, used a Norwegian discovery sample and replicated the findings in the ADNI or PING cohorts (Joyner et al. 2009; Rimol et al. 2010; Bakken et al. 2012).

Even for high frequency variants, neuroimaging databases of 1,000 subjects would be underpowered to detect commonly observed effect sizes. We must bear in mind that, in the standard experimental design (see below for others), the significance of a genome-wide hit has to be at least 20 million to one, to account for the very large number of variants tested. Some have argued that neuroimaging studies reporting effects of candidate genes—such as *COMT* or *BDNF*—are also at risk for false positive effects, in the sense that any number of genes could have been assessed, with no way to verify whether the paper was selectively reporting the successes (Flint and Munafo 2013; Ioannidis 2005). While this problem is shared by selective reporting of results in many fields, imaging genetics is particularly at risk because of the ease of retesting the same data. Some have raised the concern that a considerable proportion of neuroimaging genetics associations, especially those found in small samples, may not replicate in subsequent analysis. Clearly, although the number of genes in the human genome is limited, an almost unlimited number of candidate genes could be still tested (Bishop 2013; Flint and Munafo 2013).

Even so, others have argued that this might be an obvious but not in itself sufficient argument for needing very large sample sizes, as it does not take into account the increase in effect sizes enabled by careful selection of phenotypes (this is an area of neuroimaging research in itself; see Table 1), nor corroborative evidence from other sets of data, nor independent replication of top hits—which is commonly performed in GWAS studies. Clearly, an alternative way to avoid false positives is to collect additional functional evidence for a variant (the “convergent approach”). A final approach is to focus on candidate variants with known molecular function, and there is a long tradition of work relating genetic variants in the monoamine neurotransmitter pathways to risk for psychiatric illness. For instance, lower hippocampal but larger amygdala volumes have been associated with the long variant of the serotonin transporter polymorphism in major depression (Frodl et al. 2004, 2008), and variants in the 5-HT_1A_ receptor gene have been related to amygdala volume in borderline personality disorder (Zetzsche et al. 2008). Variants in the Huntington’s disease gene have also been shown to affect normal brain structure (Mühlau et al. 2012).

As the field began to come to terms with the sample sizes needed to demonstrate or replicate a genome-wide hit, alliances began to form and some studies reported genetic associations with volumetric brain measures supported by evidence from more than one cohort. Stein et al. (2011) reported a variant associated with caudate volume in young and old cohorts scanned on two continents at two different field strengths. Hibar et al. (2013b) also reported a variant associated with the volume of the lentiform nucleus on MRI scans. In some of these reports, a meta-analysis approach was used, to combine the evidence for genetic association across cohorts, in a way that weights the cohorts by their sample size or error variance.

### Formation of the ENIGMA Consortium and first project (ENIGMA1; Stein et al. 2012)

**Fig. 2:**
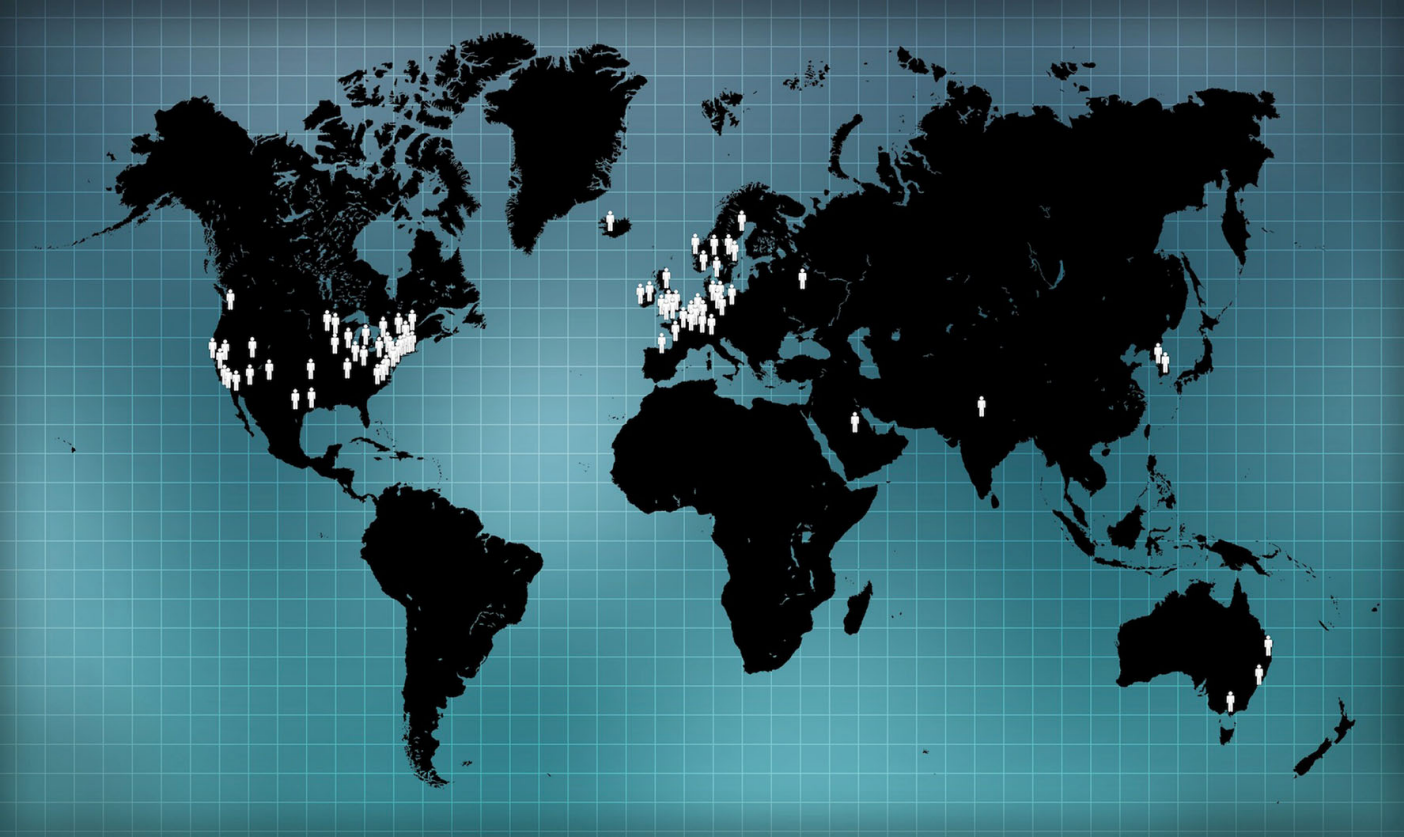
ENIGMA founding sites. The first ENIGMA project (Stein et al. 2012) was initiated in 2009, by a consortium of research groups worldwide involved in neuroimaging and genetics. Several existing consortia and research networks are taking part, including IMAGEN, EPIGEN, SYS, FBIRN, and ADNI. Many of these efforts pre-dated ENIGMA and continue today; each conducts its own projects in addition to their collaborative work within ENIGMA. ADNI collects data at 58 sites around the U.S.; for clarity, not all data collection sites are shown here. Each *symbol* represents a site contributing to ENIGMA, as of June 2013

In December 2009, a group of researchers expert in large-scale neuroimaging or large-scale genetics studies formed a network called “Enhancing NeuroImaging Genetics through Meta-Analysis” or ENIGMA. The goal of the effort was to bring together researchers with genome-wide data and images, to meta-analyze evidence in neuroimaging datasets worldwide (see Fig. 2).

**Fig. 3:**
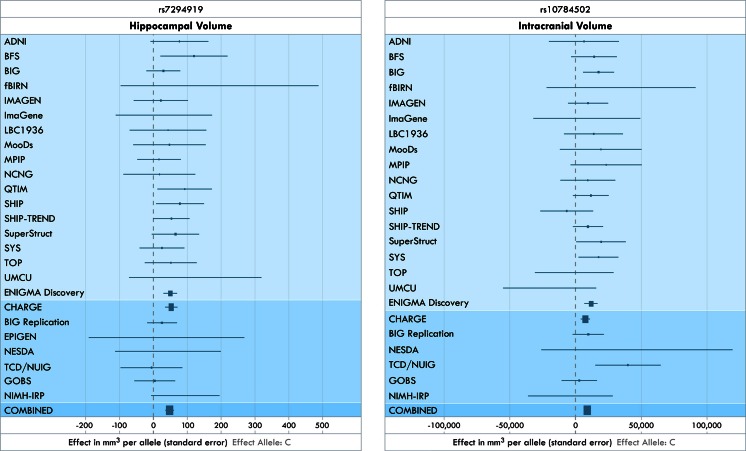
Forest plots from the ENIGMA1 study (adapted from Stein et al. 2012). Forest plots are a graphical display designed to illustrate the relative strength of an effect in different cohorts. In the *left panel*, we show the effect of the genetic variant at rs7294919 on the hippocampal volume, in a range of cohorts in ENIGMA. In ADNI, for example, the confidence interval on the effect overlaps zero, which means that there is no evidence to reject the hypothesis of no effect, if only that cohort were considered. The “ENIGMA Discovery” line combines the effects of all cohorts above it. At the bottom of the figure, the meta-analysis of all effects above the line includes data from another large consortium, CHARGE, and several replication samples. The area of each square is proportional to the study’s weight in the meta-analysis. The *right panel* shows a similar plot for the effect on intracranial volume of the common genetic variant at rs10784502. It is not necessary for the effect to be detected in all cohorts for the meta-analysis to support the effect. The abbreviations denote the names of the different cohorts in ENIGMA (please see Stein et al. 2012, for details). [Adapted, with permission, from Stein et al., *Nature Genetics,* April 15 2012]

In Stein et al. (2012), the ENIGMA consortium, in collaboration with another multi-site consortium (CHARGE; Seshadri et al. 2010; Fornage et al. 2011; Bis et al. 2012; Ikram et al. 2012) reported that the mean bilateral volume of the hippocampus was significantly associated with the intergenic variant rs7294919 (see Forest plots, Fig. 3).

The genotype at that locus was also nominally associated with expression levels of the positional candidate gene *TESC* in adult human temporal lobe tissue, based on the UCL database of brain tissue resected during epilepsy surgeries. *TESC* is currently not well studied, but it is known that it is expressed during brain development in mice and chickens (Bao et al. 2009). It encodes tescalcin, which interacts with the Na^+^/H^+^ exchanger (NHE1) (Baumgartner et al. 2004), important in the regulation of intracellular pH, cell volume and cytoskeletal organization. In addition, intracranial volume was significantly associated with a variant (rs10784502) in the *HMGA2* gene that had been previously tied to height. The CHARGE consortium had focused on elderly cohorts recruited primarily for studies of cardiovascular health in old age, whereas ENIGMA had aggregated data from cohorts across the lifespan showing the genetic effect was strong, happening over multiple stages of brain growth. Both efforts had strengths—the number of cohorts in ENIGMA (23) was larger than that of CHARGE, but some of the constituent cohorts in CHARGE numbered over 2,800 individuals.

### Practical considerations and opportunities

The first ENIGMA project demonstrated the feasibility of discovering statistically significant effects of “single letter” genomic differences in brain data worldwide, using imaging and genetic data collected using diverse protocols. Novel, harmonized data analysis and meta-analysis protocols were vital to the success of this project.

First, all genotypic data was imputed to the HapMap3 reference dataset to correct for diversity of the genotyping chips (this reference was subsequently updated to the 1,000 Genomes dataset for ENIGMA2, which began in May 2012). The imputation protocols (detailed at enigma.ini.usc.edu) and the standard reference datasets allow the consistent reporting of genotypes at the same set of genetic loci across cohorts. Imputation effectively adds prior knowledge to the data and may in this way also increase the power of a study. Not all ENIGMA cohorts are Caucasian: the GOBS cohort consists of Mexican-Americans and the NIMH cohort contains a significant number of African-Americans. As in any GWAS, population structure is taken into account during the statistical modeling of associations, to ensure that differences in SNP frequencies with ancestry are not picked up as spurious associations. Also in the imputation step, the appropriate reference populations are used for each individual, which may in some cases be Yoruban or Hispanic, as well as the CEU population that is used to represent Caucasians. However, it is not a computationally trivial task to impute very large samples of data from so many subjects. The successive refinement of reference panels means that re-imputing the same dataset to the most current standards is likely to further boost power.

Second, the exchange of data between the ENIGMA and CHARGE consortia, for the Stein et al. (2012) study, involved a reciprocal look-up rather than an exchange of the full meta-analyzed data across the entire genome. This effort found that the top hits of both consortia were in fact the same ones—providing extremely high credibility to the hits. Even so, the full genomic data were not exchanged or meta-analyzed; this more powerful effort is currently underway.

Third, the effort to harmonize the analysis of imaging data involved development of new quality control procedures. Considerable time was devoted to checking outliers, testing of the histograms and statistical distributions of brain structure volumes, and checking the allele frequencies, genomic inflation factors, and other statistical summaries of the cohort data. The genetic and MRI analysis protocols used in ENIGMA2, including quality control steps, are available online, at http://enigma.ini.usc.edu/ongoing/gwasma-of-subcortical-structures/ and the DTI analysis protocols are available at http://enigma.ini.usc.edu/ongoing/dti-working-group/.

In a project of such a scale, the need for adequate data curation is paramount. Several measures were assessed to determine whether structures had values in the expected range for volume, hemispheric asymmetry, etc. In the end, the large number of contributing authors and contributing sites made it easier to identify sites that had outliers in their genotypic or brain measurement data, partly because normative data were becoming available from all the other sites. All relevant demographic factors were controlled for at each site, including population structure that might lead to ancestry differences masquerading as true genetic associations to brain traits.

Among these tests, we demonstrated that different, widely-used programs to measure regional brain volumes produced consistent results on a broad range of cohorts worldwide, based on data from young and old samples and mixtures of both (Stein et al. 2012; see supplement comparing FSL and FreeSurfer segmentations). It was not feasible to require all sites to use one specific program to measure brain regions; as might be predicted, no single algorithm performed best for quantifying brain volumes across all datasets.

Fourth, ENIGMA did not use the “mega-analysis” model adopted by the Psychiatric Genomics Consortium, where all phenotypic and genotype data are sent to a centralized site for analysis. Instead, ENIGMA uses a “meta-analysis” concept: GWAS was run locally using pre-agreed covariates—often with and without adjustments that may affect interpretation (e.g., adjustments for overall head size). Subsequently, and after quality control at each site, the *p*-values and regression coefficients were combined and meta-analyzed in a way that weights the results based on the sample sizes of each contributing cohort.

Many cohorts within ENIGMA have restrictions on data use and data access. Some preclude the sending of data out of the lab where the data were collected. Some restrict the sending of personally identifying information to any other site. A founding goal of ENIGMA was to not require cohort data to be shared outside the center that collected it, to avoid creating ethical and legal issues for the study sites. Although data sharing is a laudable goal in science, a more pragmatic compromise has been to send protocols to distributed sites and analyze summaries of the resulting data that lack personally identifying information. This also encourages maximum participation as each site retains fiduciary responsibility for their data and its curation and integrity. Nevertheless, there is a growing perception of the community that data sharing is one fundamental building block of reproducibility in science, and a rapidly expanding number of imaging datasets are being shared thus facilitating discoveries. New ways of acknowledging data acquisition are being developed concurrently (Poline et al. 2012).

However, there are also disadvantages associated with the meta-analysis approach. For instance, it is more challenging to perform meta-analyses for more complex and potentially more informative analyses such as (1) polygenic scoring, which determines how much of the phenotypic variance can be explained by common SNPs in aggregate (Purcell et al. 2009), (2) structural equation modeling to demonstrate evidence for the endophenotype concept (Kendler and Neale 2010), and (3) stepwise linear or ridge regression, to identify the causal variant at an associated locus (McCarthy et al. 2008; McCarthy and Hirschhorn 2008). Not being able to perform interaction analyses (e.g., disease by SNP) is also a limitation, although this could be overcome as ENIGMA’s disease related working groups expand.

In the aftermath of ENIGMA1, several investigators requested access to the GWAS meta-analyzed results, and the meta-analyzed data were made available online through an interactive website named ENIGMA-Vis (Novak et al. 2012; http://enigma.ini.usc.edu/enigma-vis/). This interface allows a user to input any gene (or SNP) that they are interested in, and query its effects on a wide variety of brain measures. The current dataset also allows targeted studies of individual genes, enabling research groups, even if they are not involved in human studies, to assess the possible impact on the brain of genes they are studying (via the ENIGMA-Vis search tool).

In one study, Bulayeva and colleagues (Bulayeva et al. 2013) performed a linkage analysis of psychosis and mental retardation in population isolates in remote areas of Dagestan and Chechnya. They were able to implicate the top hits in ENIGMA1 in these illnesses, supporting a psychiatric effect of the genetic variant in human populations. Erk et al. (2013) also studied rs7294919—ENIGMA1’s top hit for association with hippocampal volume, and found that it related to behavioral differences and different patterns of brain activation in memory tasks. Work by Ming Li and colleagues (Li et al. 2013) also found SNPs in the candidate gene *CREB1* that were associated with bipolar disorder (the most significant of which was rs6785) and were also associated with measures of hippocampal volume and function.

### Current projects of the ENIGMA Consortium

ENIGMA2 is a follow-on study from ENIGMA1, in which the volumes of all major subcortical structures are subjected to genome-wide association analysis (Hibar et al. 2013a). These volumes are known to be moderately to highly heritable, with one recent study reporting highest heritability estimates for the thalamus (0.80) and caudate nucleus (0.88) and lowest for the left nucleus accumbens (0.44; den Braber et al. 2013). The structures are also implicated in a wide range of psychiatric and degenerative brain disorders, making it crucial to identify genetic and environmental factors that may influence them. Preliminary meta-analysis is underway which will be followed by functional characterization of the key hits, with a full report to be submitted soon. A range of functional evaluations is envisaged over the short and long term, ranging from *in silico* assessments of eQTL data (for instance), all the way to new functional experiments in neuronal cell lines or animal models. This kind of follow up study is crucial, given the difficulties of obtaining robust functional variants from GWAS studies.

### ENIGMA-CHARGE genome-wide meta-analysis

Researchers from the ENIGMA and CHARGE consortia have recently joined forces to perform a genome-wide meta-analysis of hippocampal and intracranial volume using updated versions of the data analyzed in ENIGMA1. Specifically, both CHARGE and ENIGMA will be meta-analyzing GWAS results from the much more densely sampled 1,000 Genomes reference set; rather than exchanging only the top hits, the entire genome-wide data is being meta-analyzed. In addition, the ENIGMA project will be contributing updated results from many new samples who have joined the effort since the completion of the ENIGMA1 project. This analysis is currently ongoing.

ENIGMA-DTI is a Working Group developing a harmonized protocol for analyzing DTI data for GWAS meta-analysis (Kochunov et al. 2012). Diffusion tensor imaging offers a range of measures that reflect the microstructure of both white and gray matter (by probing the diffusion profile of the water molecules). It is also possible to reconstruct macroscopic structures such as tracts, using the tensor’s directional information. Understanding the genetic factors underlying the connections of the brain is one of the most challenging projects in the imaging genetics field. This is especially true since the inherent plasticity of the developing brain allows for the remodeling of connectivity based on environmental influences. Interestingly though, heredity of diffusion weighted measures remains quite high, suggesting that factors related to brain growth, development, and plasticity are also highly heritable (White et al. 2013).

**Fig. 4:**
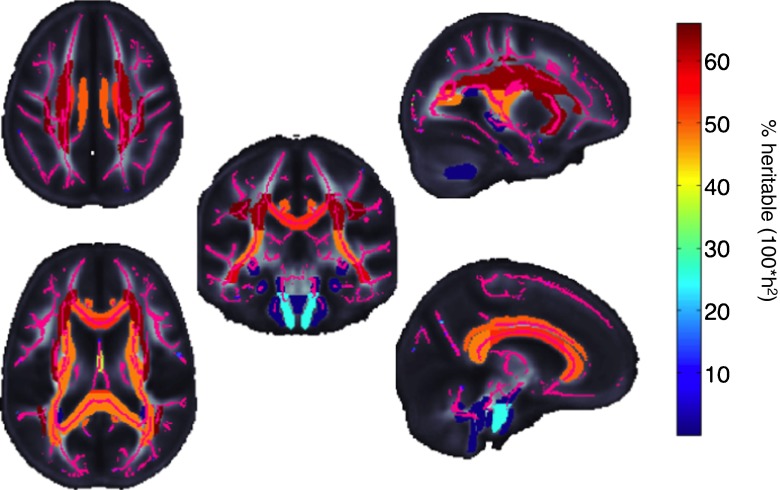
A meta-analysis of tract-wise heritability, by the ENIGMA-DTI working group, showed most tracts in the brain are moderately to highly heritable across cohorts of different ethnicities, even though they were imaged with different parameters. The “skeleton” of the white matter, reconstructed using a widely used DTI analysis program called “tract-based spatial statistics” (TBSS; Smith et al. 2006), is shown in purple for reference. The corticospinal tract (*in light blue*) was the least heritable region of interest and therefore, will not be carried forward as a phenotype in an initial GWAS of DTI-FA measures. Other methods for phenotype selection and prioritization are summarized in Table 1

A major theme in prioritizing brain phenotypes for large-scale genetic analyses is the presence of significant heritability, indicating that a proportion of individual variance in the phenotypes can be explained by genetic variation. The effect size of individual variants cannot be inferred from the heritability and higher heritability does not translate to a higher likelihood of a positive GWAS finding. However, phenotypes whose heritability is not significantly different from zero may not be good candidates for GWAS analysis because of the lack of variance due to additive genetic factors. Jahanshad et al. (2013a) screened a number of regions of interest across multiple cohorts, finding high heritability for most major white matter pathways and consistent heritability across pedigree-based and twin-based samples (Fig. 4; Kochunov et al. 2012; Jahanshad et al. 2013a). A variety of DTI parameters could be measured reliably among individuals, and genetic factors explained a substantial proportion of the variance for the most commonly used DTI measures.

Penke et al. (2010) showed strong correlations among measures of DTI-derived fractional anisotropy (FA) from a range of major white matter tracts in the Lothian Birth Cohort 1936 (LBC1936), a group of community-dwelling subjects in their seventies. Applying principal component analysis, a general white matter integrity factor was found that explained around 45 % of the individual differences in FA across eight tracts, and was significantly correlated with processing speed. Lopez et al. (2012) conducted a GWAS on this general white matter integrity factor on 535 subjects of the LBC1936 study and found suggestive genome-wide association with SNPs in *ADAMTS18* and *LOC388630*. Initial studies suggest that a proportion of the variance in fiber integrity can be predicted from common variants (Kohannim et al. 2012; Jahanshad et al. 2012, 2013c; Thompson and Jahanshad 2012; Braskie et al. 2012; Sprooten et al. 2013).

Genome-wide screens of connectome data have also been successful, and have shown the benefit of ranking connections by their heritability, prior to entering the genome-wide screening phase (Jahanshad et al. 2013b; Thompson et al. 2013). ENIGMA has not yet attempted a multi-site genetic study of brain connectivity. Agreement on a protocol depends on ongoing harmonization of DTI analysis across ENIGMA sites, which is being finalized by the ENIGMA-DTI working group (Jahanshad et al. 2013a). The harmonized DTI analysis will also allow a multi-cohort examination of the association between white matter integrity and general cognitive ability across the life course from late adolescence to old age (see also Penke et al. 2012). Also underway is a harmonization of cortical segmentation, which is being studied empirically by the ENIGMA working groups that focus on psychiatric illness (see below).

### ENIGMA Disease Working Groups

An implicit goal of ENIGMA is to see whether genetic variants impact the brain in a way that affects disease risk. In fact, a number of imaging genetics papers have identified genetic variants that appear to affect the brain and behavior; Hall et al. (2006) and McIntosh et al. (2008) reported a variant in the neuregulin 1 gene that was associated with abnormal cortical function, altered white matter integrity, and with psychosis, and there are many other examples of variants with wide-ranging effects.

Around a third of the data in ENIGMA is from patients with psychiatric illness, so once ENIGMA1 was complete, a large volume of new data could be brought to bear on the question of brain differences in a variety of disorders. To make connections between psychiatric risk genes and brain measures, it may make sense to prioritize brain measures with robust case–control differences. Of course, robust case–control differences do not, in themselves, imply that the same genetic variants influencing the phenotype will be the same as those associated with disease risk. A more informed way to rank brain measures for genetic screening is to use the endophenotype ranking value (ERV; Glahn et al. 2012), which aims to rank biomarkers in terms of their promise as endophenotypes for any heritable illness.

The ERV balances the strength of the genetic signal for the endophenotype and the strength of its relation to the disorder of interest. It is defined using the square-root of the heritability of the illness (*h*_i_^2^), the square-root of the heritability of the endophenotype (*h*_e_^2^), and their genetic correlation (ρ_g_):

$$ {\mathrm{ERV}}_{\mathrm{i}\mathrm{e}}=\left|\surd {h_{\mathrm{i}}}^2\kern1em \surd {h_{\mathrm{e}}}^2{\rho}_{\mathrm{g}}\right|. $$

**Fig. 5:**
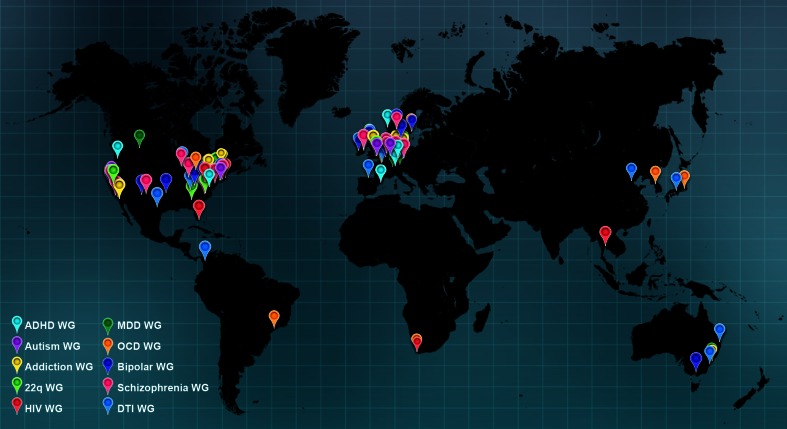
Locations of the ENIGMA Working Groups. After ENIGMA’s first project was completed (ENIGMA1; Stein et al. 2012), large amounts of brain imaging data had been analyzed from patients with a variety of psychiatric disorders. Working groups (WGs) were formed to understand the effects on the brain of bipolar disorder, major depressive disorder (MDD), schizophrenia, by pooling and comparing data from many neuroimaging centers. These groups are open to any researchers who have collected MRI scans from patients with these illnesses. No genetic data is needed to join. In fact, most projects study factors that might influence how these disorders affect the brain—medications, geographic factors, and the age and gender of the patient. A further working group focuses on diffusion tensor imaging, which assesses white matter integrity; current projects relate DTI measures to individual differences in cognition and genetic make-up. The institutions in the working groups, as of June 2013, are shown on the map (*see color key, inset*)

In schizophrenia, for example, decades of studies have reported morphometric differences in patients versus controls, for a range of different structures. ENIGMA offers a promising framework to rank brain measures in order of their effect sizes for case–control differences; these effects could also be further weighted based on their genetic correlation with the illness, to give another ranking. Nor is it expected that the disease should have identical effects on the brain in all cohorts; the variety of participating cohorts in ENIGMA makes it possible to dig deeper into medication-related, or even geographic or demographic factors to explain why brain differences vary so drastically across different studies (see Fig. 5 for the locations of ENIGMA sites in disease-related working groups).

In a meta-analysis of data from 1,136 patients and 1,401 controls, the ENIGMA-Schizophrenia Working Group found that hippocampal volume gave among the highest effect sizes for any subcortical brain structural difference in schizophrenia (Turner et al. 2013; van Erp et al. 2013). In a related meta-analysis of structural MRI data from 1,022 patients and 1,415 controls, the ENIGMA-Bipolar Disorder Working Group found consistent differences across the subcortical regions, but in a different pattern than that characteristic of schizophrenia (Hibar et al. 2013a). There were significant reductions in the bilateral thalamus, hippocampus, and amygdala in patients diagnosed with bipolar disorder. In general, the trend revealed a decrease in subcortical volumes throughout the brain in patients with bipolar disorder. This work is clinically important, as the alterations in limbic and some cortical regions are thought to underlie some of the affective symptoms in bipolar disorder; even so, the source of many of the subcortical and cortical differences in the disorders has been a matter of debate, and for many structures, morphometric findings have not always been consistent. This work is still ongoing, with a total sample of 4,729 subjects from 15 cohorts worldwide (2,060 patients and 2,669 controls, as of November 2013).

In addition, following the model of the Schizophrenia and Bipolar Disorder and working groups, an ENIGMA-Major Depressive Disorder (MDD) Working group was recently initiated. It already includes structural MRI data from 1,850 patients and 3,483 controls. Data analysis is currently in progress.

An additional working group on attention deficit/hyperactivity disorder is currently being formed, ENIGMA-ADHD, which will analyze data from more than 1,500 cases (children and adults) with the disorder and over 2,000 controls.

An even more recent extension is a developing ENIGMA-Addiction working group that will analyze data relevant to addiction phenotypes including case-control comparisons across a variety of substances, and going beyond this to examine the influence of comorbidities, gender and stages of disorder. The ENIGMA meta-analytic approach will be used to aggregate data from case-control and developmental cohorts to examine the relative contribution of various genetic and brain correlates to risk for early onset substance misuse, transition to regular use, susceptibility to dependence, and individual differences in relapse vulnerability.  We will also examine variants common to all addictive behaviors, and attempt to identify those that might be specific to homogeneous addiction profiles.  To date, a pooled sample of 8,000 participants with neuroimaging, genetics and addiction phenotypes has been identified; their data will be re-analyzed to address these questions.

Clearly, these studies aggregate data from cohorts that are heterogeneous in terms of duration of illness, disease etiology, medication history, demographics, exposure to potentially neuroprotective substances such as lithium, and many other factors. But part of the richness of the ENIGMA efforts is that they afford sufficient power to begin to see which of these factors—including geographic factors, perhaps—affect disease expression in the brain and the universality, or otherwise, of the biomarkers of brain disease. Based on these findings, a key direction for ENIGMA is to see how genes that affect the brain relate to genes that affect risk for psychiatric illness (identified by the Psychiatric Genomics Consortium, https://pgc.unc.edu/), to identify shared biology between brain characteristics and disease. A collaboration between ENIGMA and the PGC is now underway to study these relationships.

One limitation of ENIGMA to date is that the sites contributing to the meta-analysis include a clinically somewhat heterogeneous group of population-based studies, and case–control cohorts from multiple psychiatric and neurological diseases. ENIGMA’s working groups on psychiatric disorders are meta-analyzing representative datasets from schizophrenia, bipolar, depressed, ADHD, autism, OCD, 22q deletion syndrome, HIV, addiction, and other cohorts worldwide. Eventually, cross-disorder comparisons should be able to identify common and distinctive patterns of brain morphometry, and how they depend on genetic variation, and diagnostic criteria. Psychiatric cohorts vary in their inclusion and exclusion criteria, and in duration of illness, medication history, ethnicity, and in other demographics. Some studies include patients with co-morbid conditions deliberately excluded by other studies. The diversity of psychiatric cohorts in ENIGMA suggests a second wave of analyses to understand cohort-specific factors that might account for, or contribute to, the heterogeneity in results across sites. Recent work by the Psychiatric Genomics Consortium Cross-Disorders working group has identified considerable genetic overlap between several major disorders, at the level of common genetic variants (Lee et al. 2013). ENIGMA may be able to do the same from a neuroimaging perspective, to determine if genetic factors implicated in different disorders account for some of the cross-disorder differences in the brain imaging meta-analyses.

### Future directions and caveats

Perhaps the most exciting strength of ENIGMA is its ability to unite researchers using neuroimaging worldwide in a common purpose. The fact that so many investigators are actively involved makes it possible to benefit from the combined resources and talents of all participants for “crowd-sourcing” discovery. Also, the sample sizes involved—unprecedented for a neuroimaging study—alleviate some of the concerns about underpowered studies and unreliable findings (Button et al. 2013). In addition, apart from identifying genetic variants, another important role for the ENIGMA consortium is to help understand how GWAS-derived genetic variants for behavioral phenotypes influence the brain. Exploring the effects of disease risk alleles on brain measures can help us understand the brain systems affected, and at which stage—and also whether the effects are pervasive or selective (de Geus 2010).

Much of this overview of the history and future efforts of ENIGMA highlights its relevance to studies of disease, focusing on psychiatric and neurodegenerative disorders such as Alzheimer’s disease. Even so, only around a third of the ENIGMA data comes from patients with psychiatric illness, and much can be learned about the genetic factors that drive normal variation in the general population. A great deal of fundamental information on the biology of the human brain can be discovered from efforts such as ENIGMA, irrespective of whether it has a direct relevance to any specific disease.

There are limitations to a study like ENIGMA despite its strengths. The first is that many other types of genetic or epigenetic variation other than GWAS are important—rare variants, CNVs, expression and methylation analyses are all crucial; they simply have not yet been evaluated through ENIGMA, but that is likely to change in the future. In recent genome-wide complex trait analyses (“GCTA” analyses; Yang et al. 2011; Lee et al. 2011), Wray, Visscher and their colleagues have shown that GWAS data may account for a surprisingly high proportion of genetic variance in a trait, even when the individual predictive value of a given locus or SNP is low.

As a basic principle of genetics, an overall large heritability does not guarantee locus specific heritability, but recent discoveries have surprised some geneticists in supporting the explanatory power of SNPs. For example, despite early results that accounted for about 5 % of the variance in height, large studies have now demonstrated SNPs can account for around 45 % of the variance in height (for which the overall heritability is around 80 %). Also, common causal variants may account for around 23 % of the risk for schizophrenia (Lee et al. 2013) and up to 60 % of the risk for autism (Klei et al. 2012). Given the polygenic architecture of these disorders, these results suggest that more individual SNP associations will be detected for each disorder, as sample sizes increase.

Some authors have emphasized that similar kinds of “polygene” scores to the ones used to predict illness risk can be used to explain variation in related phenotypes, such as cognition (Davies et al. 2011), structural MRI (Holmes et al. 2012), neural activation on functional MRI (Whalley et al. 2013a) and white matter integrity (Whalley et al. 2013b).

Even so, some geneticists argue that rarer variants are in some cases more worthy of study than GWAS as they tend to have larger effects that are more easily validated functionally. GWAS has been successful when used for QTL localization, but the over-arching goal of complex disease genetics is to identify the causal genes and functional variants responsible for phenotypic variation. So some have argued that GWAS is useful for detecting the signal from common variants, but that few GWAS results have turned into functional variants or genes (across all disease domains). In principle, ENIGMA could be used to study rare variants as well, but a range of complementary approaches are always necessary.

Second, the geographical diversity of ENIGMA is higher than that of most neuroimaging studies, but there may be important ethnic differences in allele frequency that complicate the generalization of results to all human populations. This limitation is not specific to ENIGMA, and its consortium structure could be expanded geographically to assess ethnic differences in factors associated with disease and their relationship to brain structure. Ethnic differences in genetic effects are well known: the meta-analysis of the effects of the *APOE* risk gene for Alzheimer’s disease shows a difference between European and Asian populations. So far, people in Africa and Asia are under-represented on the ENIGMA map; we are therefore keen to include samples from these continents.

Third, the need for multivariate analysis has long been known in the neuroimaging field—no serious neuroscientist would predict diagnosis from a single pixel or voxel in an image—the multivariate pattern of signals is paramount. Methods to access and recover the maximum amount of pertinent information in an image dataset are only just in their infancy in the fields of genetics and neuroimaging—partially due to the lack of sufficient data to test competing methods, until recently (Liu et al. 2009; Stein et al. 2010; Hibar et al. 2011b; Vounou et al. 2010; Le Floch et al. 2012; Rosenblatt et al. 2013; Meda et al. 2010, 2012).

Fourth, the discovery of a GWAS hit—in ENIGMA or any other GWAS study—is the beginning of a long road of discovery, especially if the finding is intergenic or in a gene of unknown function. Some genomic screens of anatomical or structural connectivity data have implicated genes such as *SPON1* (Jahanshad et al. 2013b) and *FRMD6* (Ryles et al. 2012) that were discovered in later case–control studies to be risk genes for AD (Hong et al. 2012; Sherva et al. 2013). Functional validation of genetic variants reliably implicated in large scale studies will be the way we learn new biological processes and further our understanding of risk for psychiatric diseases.

Fifth, ENIGMA has started by analyzing those phenotypes that are easily measured in a standardized way. Brain images can be analyzed in more sophisticated ways than traditional morphometric methods; shape analysis, for example, has long been used to characterize features of brain structure, including cortical complexity, curvature, fractal dimension, spectral content, and other indices. Also, the reporting of regional summaries from DTI data clearly does not exploit all of the available information in the data—DTI can be analyzed using automatic whole-brain tractography to reveal the brain’s fiber patterns and measure connectivity, and even the topology of these brain networks. Despite the ever-expanding landscape of brain features that can be studied, the large samples required for genetic analysis have motivated the study of the simplest brain measures first. Undoubtedly the future will hold large scale genetic studies of brain connectivity, anatomical shapes, functional networks, and features that are as yet unknown and undiscovered.

As a final limitation, ENIGMA analyses have been cross-sectional to date, rather than longitudinal. Genetic predictors of brain changes over time have substantial importance for clinical trial enrichment (e.g., *TREM2*, which harbors variants that predict rates of brain atrophy over time; Rajagopalan et al. 2013; see Kohannim et al. 2013, for an approach to boost power in drug trials by multi-locus genetic profiling; see Andreasen et al. 2012, for an approach examining epistatic relationships between multiple genes and progressive brain tissue loss in schizophrenia).

### Are fewer subjects needed with imaging?

An open question is whether GWAS meta-analyses really do require fewer subjects with imaging than they do when behavior is the target of study. In 2009, before ENIGMA began, one of its founders noted, “Just because the phenotypes are expensive to collect does not change the power calculations.” (N. Martin, pers. commun., 2009). But more recently, Rose and Donohoe (2013) performed an empirical analysis of effect sizes in genetic studies of cognitive and neuroimaging traits in schizophrenia, and found evidence supporting the efficiency of using imaging traits. However, some evidence does suggest that imaging traits may have intermediate effect sizes when compared to phenotypes theoretically closer or farther away from the underlying biology. The percent variance explained in gene expression GWASs (often called eQTL studies) for the top SNP hits are well above 10 % of the variance in the expression of a particular gene (Stranger et al. 2007). The percentage of trait variance in hippocampal volume explained by the top genetic variant in ENIGMA1 was 0.27 % (Stein et al. 2012) although independent cohorts will be required to estimate the explained variance in the population at large. Finally, the top hit in one genome-wide analysis of traits correlated with cognition—such as educational attainment—explained 0.02 % of the variance (Rietveld et al. 2013).

It is interesting to compare the effect of ENIGMA’s top SNPs with the effects of common psychiatric and neurological conditions on hippocampal volumes. In a recent meta-analysis (Barnes et al., 2009), patients with Alzheimer’s disease have a 24.1 % mean hippocampal volume deficit. Patients with mild cognitive impairment have a 15.3 % deficit relative to cognitively healthy controls (calculated from Table 9 in Leung et al. 2010). In psychiatric disorders, the hippocampal volume deficit is typically reported to be smaller. Although hippocampal volume deficits are one of the more robust MRI findings in schizophrenia, they are not always evident in single site studies (Shenton et al. 2001). The ENIGMA Disease Working Groups now suggest a mean hippocampal volume deficit, relative to controls, of around 3.6 % in schizophrenia and 2.9 % in bipolar disorder—depending, of course, on cohort-specific factors (medication, duration of illness, etc.). By contrast, in ENIGMA1, the rs7294919 genetic variant was associated with a hippocampal volume decreased by 47.6 mm^3^ or by 1.2 % of the average hippocampal volume per risk allele. Bearing in mind that the cause of the effects is quite different, the effect of ENIGMA’s top hippocampal SNPs on hippocampal volume is approximately 5 % of the mean effect of Alzheimer’s disease, around one-third of the effect of schizophrenia, and around 40 % of the effect of bipolar disorder. If such effect sizes are typical, sample size requirements will generally be larger in genetic association studies than in neuroimaging studies of disease effects on the same phenotypes, but not vastly larger.

To some researchers, these preliminary observations suggest an expected pattern of effect sizes, whereby GWAS for cognitive traits may have top hits with smaller effect sizes than those for imaging traits, which in turn may tend to have smaller effect sizes than expression traits. Also, now that consistent hits have been identified in ENIGMA1 and ENIGMA2, it should be possible to estimate effect sizes for the same hits in new replication samples, to understand what sample sizes are sufficient to detect them. Conversely, some have argued that the available data on genetic effect sizes at different levels of neuroscience are currently too limited to draw conclusions from only the three data points cited here. Clearly, we do not yet have sufficient information to say whether imaging genetics effects sizes will always be larger than effect sizes for cognitive/behavioral phenotypes. One way to begin to address this question might be to quantify effects of some specific functional SNP at three different related levels, such as eQTL data from the hippocampus, hippocampal volume on MRI, and a behavior that is tightly dependent on hippocampal function; this may show the appropriate differential effect sizes in one framework.

A further interesting angle is the follow-up of the top ENIGMA1 hits in ethnically isolated cohorts of Dagestan and Chechnya by Bulayeva et al. (2013). Genetic isolates can be valuable for studying any human phenotypes; required sample sizes for studying genetic effects can be smaller than in heterogeneous outbred populations due to the genetic homogeneity of these isolates. This has long been noted by classical statistical and population geneticists (e.g., Falconer 1960; Neel 1992; Bulayeva et al. 2005). As genetic isolates have a high rate of traditional endogamy and inbreeding, it is not possible to perform GWAS, but genome-wide linkage analysis is possible (Sheffield et al. 1998). In particular, the analysis of isolated populations makes it less challenging to study polygenic disorders by reducing the number of loci possibly involved in the disorder.

ENIGMA now focuses on genetic analysis of neuroimaging measures, but psychiatric diagnosis is important as well. ENIGMA does not simply relate imaging data to genetics; many of its working groups study the relationship between imaging measures and diagnosis. So, in a sense, psychiatric diagnosis is also a key target of study. For example, recent GWAS and follow-up studies have provided strong statistical evidence that variation in the *NCAN* gene is a common risk factor for bipolar disorder and schizophrenia (Cichon et al. 2011; Mühleisen et al. 2012). Both studies found that the A allele of SNP rs1064395 is a risk-mediating allele and that rs1064395 influences risk to a broad psychosis phenotype. To identify a putative mechanism, Schultz et al. (2013) tested whether the risk allele has an influence on brain structure. In patients with schizophrenia, they found a significant association with higher folding in a right lateral occipital region and at a trend level for the left dorsolateral prefrontal cortex. Controls did not show an association. The findings suggest a role of *NCAN* in visual processing and top-down cognitive functioning. Both major cognitive processes are known to be disturbed in schizophrenia. Another GWAS study by Rietschel et al. (2012) identified a risk factor for schizophrenia in a chromosomal region harboring the genes AMBRA1/CHRM4/DGKZ/MDK (rs11819869). In an independent follow-up analysis, they found that healthy carriers of the risk allele T showed altered activation in the subgenual cingulate cortex during a cognitive control task. This brain region is a critical interface between emotion regulation and cognition, which are structurally and functionally abnormal in schizophrenia and bipolar disorder.

The recent successes of psychiatric GWAS have unearthed a vast resource of findings when sample sizes became very large (Ripke et al. 2011, for the Schizophrenia Psychiatric Genome-Wide Association Study (GWAS) Consortium; Sklar et al. 2011, for the Psychiatric GWAS Consortium Bipolar Disorder Working Group; Cichon et al. 2011, for the MooDS consortium Bipolar Disorder Group; Rietschel et al. 2012, for the MooDS consortium Schizophrenia Group; Cross-Disorder Group of the Psychiatric Genomics Consortium et al. 2013). In fact, it was the need for large samples that encouraged the ENIGMA groups to work together, realizing that otherwise their power to make credible discoveries in genomic scans would be severely limited.

A second question is which of the recently reported GWAS findings are true or credible if they were discovered in small cohorts. ENIGMA focuses on meta-analyses, but some have argued that smaller individual studies—when replicated—may discover interesting (and strong) associations. Paus et al. (2012) noted that they had run a successful GWAS in only around 300 participants. Also, some GWAS signals may be important in the context of some individual cohorts, but may be washed out in meta-analyses. A more skeptical line of argument is that if we need 20,000+ samples to detect an effect, most likely what we see has very small effect size. Even so, some recent GWAS analyses appear to have picked up variants with strong effects—e.g., that roughly double disease risk (e.g., in *TREM2*), and these effects have been confirmed in smaller cohorts by comparing brain images from carriers and non-carriers (Rajagopalan et al. 2013).

One testable hypothesis is that GWAS would be more powerful and efficient if we select imaging phenotypes in a principled way; if this is true, it may be possible to perform GWAS with consistently replicated hits, that do not require tens of thousands of subjects to reject the null hypothesis. In fact, the ENIGMA working groups each aim to find heritable brain measures that maximize case–control differentiation, for studies of disease. This will help us to prioritize the future targets of study for influential genes. Although ENIGMA is data-driven, that does not mean that we cannot use patterns in the findings to design more targeted approaches that prioritize phenotypes and genetic loci for follow-up analyses.

One such analysis (Desrivieres et al. 2013) evaluates pre-selected genes that are expressed in the brain and change in their expression throughout brain development. By narrowing the search space to genes that are likely to play a role—and whose functions have more chance of being understood—the power of the study is also increased, as is its practical value for neuroscience and medicine. This must be balanced with the knowledge that approximately 88 % of GWAS hits are in intergenic regions (Hindorff et al. 2009) and almost all genes are expressed at some location in the brain at some period of the lifespan (www.brainspan.org).

ENIGMA, to date, has used a mass-univariate analysis, where each trait (or brain measure) is considered on its own, and each genetic variant is considered on its own. Recent multivariate analyses can cluster voxels in the brain—or SNPs on the genome—to empower analyses, sometimes with both forms of clustering occurring at once (Hibar et al. 2011a; Thompson et al. 2013). Some of these multivariate analysis methods have been used to detect significant hits in image-wide genome-wide searches in cohorts of under 1,000 subjects (Ge et al. 2012; Chen et al. 2012b; Jahanshad et al. 2013b). In most analyses, multivariate refers to condensing information on the imaging side, not the genetic side, although both methods and joint methods are emerging. Multivariate methods can be quite sophisticated mathematically. Some draw upon a century of powerful work in classical quantitative genetics and twin designs. Chiang and colleagues (Chiang et al. 2011, 2012), for example, computed the “cross-trait cross-twin correlation” between all pairs of voxels in an image, to pull out “image clusters” with common genetic determination (see also Chen et al. 2011, 2012a). Others have incorporated knowledge of the genetic or imaging relationships to guide the solution (Chen et al. 2013). Such tactics, among others, offer a principled way to mine high-dimensional datasets, boosting power for any subsequent GWAS.

A further line of work studies the “interactome”: it is now possible to search pairs or sets of SNPs for interaction effects in images (Hibar et al. 2013c) and some have argued that this is the norm for mechanisms of gene action, and the context of other genetic variants should be included in the analyses (Hariri and Weinberger 2003). For instance, Roffman et al. (2008) were the first to show functional MRI evidence of epistasis in schizophrenia. Their findings were consistent with epistatic effects of the *COMT* and *MTHFR* polymorphisms on prefrontal dopamine signaling, suggesting that in schizophrenia patients, the *MTHFR* 677 T allele exacerbates prefrontal dopamine deficiency. Andreasen et al. (2012) used a machine learning algorithm to identify genes/SNPs that were interacting with one another and predicting a continuous outcome measure that is a biologically meaningful phenotype (“intermediate phenotype”) for schizophrenia: changes in brain structure occurring after the onset of the illness.

Expanding the study of interactions to the whole genome, Kam-Thong et al. (2012) presented a whole genome analysis of epistasis (SNP-by-SNP) on several traits, including hippocampal volume. Pandey et al. (2012) reported a pathway analysis, which includes information from the network of gene–gene interactions as well as main effects when prioritizing genes for pathway analysis. Clearly no genetic variant acts alone; however, multiple comparisons increase exponentially with this approach implying difficulty obtaining enough power.

In conclusion, when the next round of ENIGMA studies has been completed, there will be a scaffolding of credible genetic variants on which to build and test new models of macroscale brain development, the implications of those variants for neuropsychiatric disease, and a new basis to probe the genetic architecture of the living human brain.
